# Divergent genes in gerbils: prevalence, relation to GC-biased substitution, and phenotypic relevance

**DOI:** 10.1186/s12862-020-01696-3

**Published:** 2020-10-19

**Authors:** Yichen Dai, Rodrigo Pracana, Peter W. H. Holland

**Affiliations:** Department of Zoology, University of Oxford, 11a Mansfield Road, Oxford, OX1 3SZ UK

**Keywords:** gBGC, GC bias, Genome evolution, Insulin receptor, *Medag*, Metabolism, Osteopontin, Pancreatic duodenal homeobox 1, Protein evolution

## Abstract

**Background:**

Two gerbil species, sand rat (*Psammomys obesus*) and Mongolian jird (*Meriones unguiculatus*), can become obese and show signs of metabolic dysregulation when maintained on standard laboratory diets. The genetic basis of this phenotype is unknown. Recently, genome sequencing has uncovered very unusual regions of high guanine and cytosine (GC) content scattered across the sand rat genome, most likely generated by extreme and localized biased gene conversion. A key pancreatic transcription factor PDX1 is encoded by a gene in the most extreme GC-rich region, is remarkably divergent and exhibits altered biochemical properties. Here, we ask if gerbils have proteins in addition to PDX1 that are aberrantly divergent in amino acid sequence, whether they have also become divergent due to GC-biased nucleotide changes, and whether these proteins could plausibly be connected to metabolic dysfunction exhibited by gerbils.

**Results:**

We analyzed ~ 10,000 proteins with 1-to-1 orthologues in human and rodents and identified 50 proteins that accumulated unusually high levels of amino acid change in the sand rat and 41 in Mongolian jird. We show that more than half of the aberrantly divergent proteins are associated with GC biased nucleotide change and many are in previously defined high GC regions. We highlight four aberrantly divergent gerbil proteins, PDX1, INSR, MEDAG and SPP1, that may plausibly be associated with dietary metabolism.

**Conclusions:**

We show that through the course of gerbil evolution, many aberrantly divergent proteins have accumulated in the gerbil lineage, and GC-biased nucleotide substitution rather than positive selection is the likely cause of extreme divergence in more than half of these. Some proteins carry putatively deleterious changes that could be associated with metabolic and physiological phenotypes observed in some gerbil species. We propose that these animals provide a useful model to study the ‘tug-of-war’ between natural selection and the excessive accumulation of deleterious substitutions mutations through biased gene conversion.

## Background

As animal genomes are sequenced, unexpected features are often discovered, shaping our understanding of molecular evolution and its relation to phenotypic evolution. Amongst mammals, the genomes of gerbils (order Rodentia, subfamily Gerbillinae) have emerged as some of the most unusual discovered to date. Several puzzling features of gerbil genomes are still not fully understood, and these raise important questions about mechanisms underlying molecular evolution and possible constraints or challenges to natural selection.

The first indications of unusual genome structure in this group came from analyses of the sand rat (*Psammomys obesus*), a desert-living gerbil from the Middle East and North Africa [1]. After initial attempts to clone individual genes gave misleading results [2], genome sequencing revealed presence of an ‘island’ or ‘islands’ of remarkably high GC content [3]. In the case of at least one gene located within a high GC island, *Pdx1*, these nucleotide changes are associated with unusual amino acid substitutions with potential to adversely impact physiology or development [3, 4]. Analysis of transcriptomes from additional species reveal that the ‘GC islands’ phenomenon is shared between different gerbil species and occurred in the gerbil evolutionary lineage [5].

Variation in GC content within a genome is not in itself unusual. With the increased availability of published genomes, many studies have reported differences between nucleotide composition in regions of the genome and between species [6, 7]. What marks gerbil genomes as unusual is the extreme nature of the nucleotide compositional bias, including changes within protein-coding genes. Recent work has revealed that gerbil genomes likely have one large island (~ 10 Mb) of extreme GC bias, where GC content at synonymous sites reaches almost 100%, and several other less extreme islands on other chromosomes [5]. In eukaryotes, the location of GC-rich domains is often correlated with chromosomal regions of high recombination, such as subtelomeric regions and small chromosomes [8, 9]. The correlation is thought to be driven by GC-biased gene conversion (gBGC) [10, 11], a phenomenon that occurs at meiosis when homologous chromosomes contain GC/AT heterozygous sites [8]. During strand invasion after meiotic pairing, the mismatch between strands at these sites tends to be repaired using the strand containing a G or C nucleotide in preference to the strand containing A or T [8].

This gBGC process can profoundly affect genome GC composition over generations through fixation of AT to GC (weak to strong) mutations in genomic regions with a high recombination rate [8]. This underlying process generates a pattern of GC biased evolution in these regions, with ‘weak to strong’ nucleotide substitutions occurring more often than ‘strong to weak’ nucleotide substitutions. Importantly, in genomic regions where the effect of gBGC is particularly strong, deleterious ‘weak to strong’ mutations may become fixed in the population despite the action of purifying selection [12]. Suggested examples include coding sequence changes in the *LEP* gene in the avian lineage [13], the *Fxy* gene in mice [14], and the *Pdx1* gene in sand rats and other gerbils [3]. The radical changes to the *Pdx1* gene associated with GC bias in the gerbil lineage could have physiological implications. *Pdx1* is a homeobox gene encoding a highly conserved transcription factor essential for pancreatic development and function [15–18]. In humans, *PDX1* variants are linked to pancreatic dysfunction of varying severity [19, 20], while experiments in mice have shown that *Pdx1* is essential for pancreatic development [15, 16]. Consistent with evidence from mutations, comparisons between species reveal extreme evolutionary conservation, especially in the homeodomain motif, indicating that each amino acid site is under purifying selection and that few changes are tolerated. It is therefore striking that the 60 amino acid PDX1 homeodomain, normally 100% conserved across mammals, has 15 amino acid changes in the sand rat and 14 amino acid changes in the Mongolian jird [3].

Despite the occurrence of these otherwise ‘disallowed’ mutations, sand rats and other gerbils do develop a pancreas and do secrete insulin from pancreatic β-cells [21, 22]. There are indications, however, that sand rats and possibly other gerbils are prone to disorders associated with pancreatic dysfunction under some conditions. For example, at least some sand rats develop diet-induced type 2 diabetes (T2D) when fed standard laboratory rodent food, and physiological studies have shown a predisposition towards insulin resistance and stress-induced β-cell apoptosis in T2D-prone individuals [23, 24]. In addition, some Mongolian jirds raised on a laboratory diet have also been observed to spontaneously become obese and develop poor glucose tolerance [25, 26].

We must be cautious before concluding that changes to *Pdx1*, driven by GC-biased evolution, are the cause of physiological disorders in sand rats and other gerbils. In humans, T2D is a complex metabolic disease caused by a mixture of genetic and environmental factors, with the majority of T2D cases associated with coding/regulatory sequence mutations in more than one gene [27–29]. In addition, some T2D-related genes are not clearly associated with adult β-cell function; for example, mutations in the *LEP* gene can lead to T2D by altering appetite and body weight, while mutations altering the insulin receptor gene *INSR* can cause T2D due to insulin resistance in tissues responding to insulin [30, 31].

In this study, we take a different approach to previous studies that have focused on only one protein, PDX1, or analyzed GC evolution in the gerbil genome [4, 5]. First, we focus on the gerbil proteome and we ask how many proteins, besides PDX1, have become ‘unusually altered’ in amino acid sequence during the evolution of two gerbils: sand rat (*Psammomys obesus*) and Mongolian jird (*Meriones unguiculatus*). We address this through a comparative study, searching for genes that have changed in gerbils but are conserved in other mammals. These may include genes that have accumulated potentially deleterious changes. Second, we ask if the genes encoding these unusually altered proteins in gerbils are associated with GC bias: an excess of AT to GC base pair changes. This tests whether unusual protein sequence change is being driven by the process of gBGC rather than natural selection. Third, we ask if any of the deviant proteins could feasibly be connected to propensity to type 2 diabetes or related physiological disorders. Together, these three lines of investigation combine to test whether ‘blind’ fixation of potentially deleterious mutations though biased gene conversion had consequences for the biology of these desert-living rodents.

## Results

### Many gerbil proteins are extremely divergent compared to their homologues

**Fig. 1:**
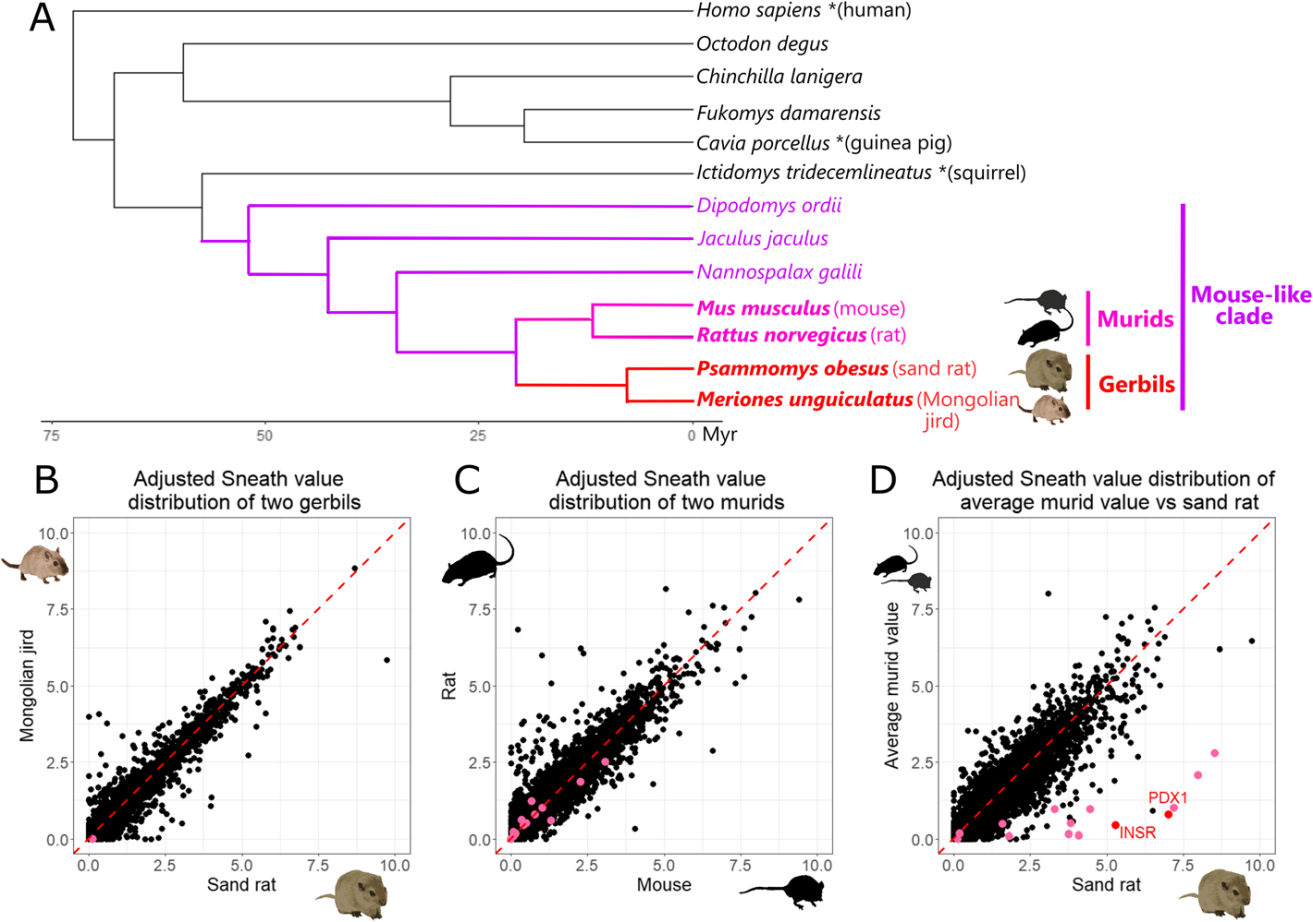
Phylogenetic relationships between species analyzed and patterns of protein sequence divergence. **a** Phylogenetic tree showing evolutionary relationships between the species analyzed, with approximate divergence time estimated by a previous study [32]. Outgroup species used for most proteins are marked with an asterisk, murid species in pink, gerbil species in red. **b**-**d** Adjusted Sneath values for orthologous proteins compared between (**b**) two gerbil species, (**c**) two murid species, (**d**) the average of two murid species and the sand rat. Each point represents one protein; proteins encoded by genes in the extreme GC-rich region of sand rat are shown in pink or (for PDX1 and INSR) in red. Only one protein encoded within the extreme GC-rich region is present in (**b**) due to missing genes in the Mongolian jird genome assembly. Photographs from J.F. Mulley and Pixabay with permission

To detect protein-coding genes that have become unusually divergent in gerbils, we use measures of ‘relative divergence’ not ‘absolute divergence’ because different types of protein evolve at different rates. High absolute sequence divergence could simply reflect low levels of selective constraint on amino acid sequence, which would be manifest as higher sequence divergence in all lineages. In contrast, high relative divergence reflects amino acid sequence changes in one species (or lineage) that is greater than the expectation for that protein. Comparing predicted proteomes from the genomes of 12 rodents, plus human as an outgroup, gave a set of 10,554 genes with one-to-one orthology between all species (Fig. 1; Additional file 2). To quantify relative divergence in amino acid sequence we used two different indices for measuring the biological and chemical relevance of amino acid changes: the Sneath Index and Epstein’s Coefficient [33, 34]. We limited analysis to positions in multiple sequence alignments that had the same residue in three nested outgroup species (usually human, guinea pig and squirrel; asterisks in Fig. 1a); this conservation suggests that these residues represent the ancestral state for rodents and they are likely functionally conserved. Some proteins had few residues meeting this criterion, reducing the dataset to 9771 sand rat genes and 10,069 Mongolian jird genes. For each sequence in two gerbil species and two murine species (mouse and rat), we scored the Sneath Index and Epstein’s Coefficient for each residue relative to that of the outgroup species. The sum across all residues gives a ‘Sneath value’ and ‘Epstein’s Coefficient’ for each protein; these are divided by the protein alignment length to give an ‘adjusted Sneath value’ or ‘adjusted Epstein’s Coefficient’.

For most genes, the adjusted Sneath value is similar between species as expected (most dots lie close to the x = y axis in Fig. 1b-d). For example, there is very high correlation between values for two gerbil species (Pearson correlation coefficient *r* = 0.973, *p* < 2.2e-16; Fig. 1b) and also between two murid rodent species (*r* = 0.943, *p* < 2.2e-16; Fig. 1c). The comparison between gerbil and murid proteins includes several outlier sequences (dots away from the x = y line), although the overall correlation is still high (*r* = 0.909, *p* < 2.2e-16; Fig. 1d). Similar results were obtained using Epstein’s coefficient: very high correlation between two gerbil species (*r* = 0.965, *p* < 2.2e-16), between two murid species (*r* = 0.924, *p* < 2.2e-16), and lower correlation between murids and sand rat (*r* = 0.884, *p* < 2.2e-16) (Additional file 1: Fig. S1).

**Fig. 2:**
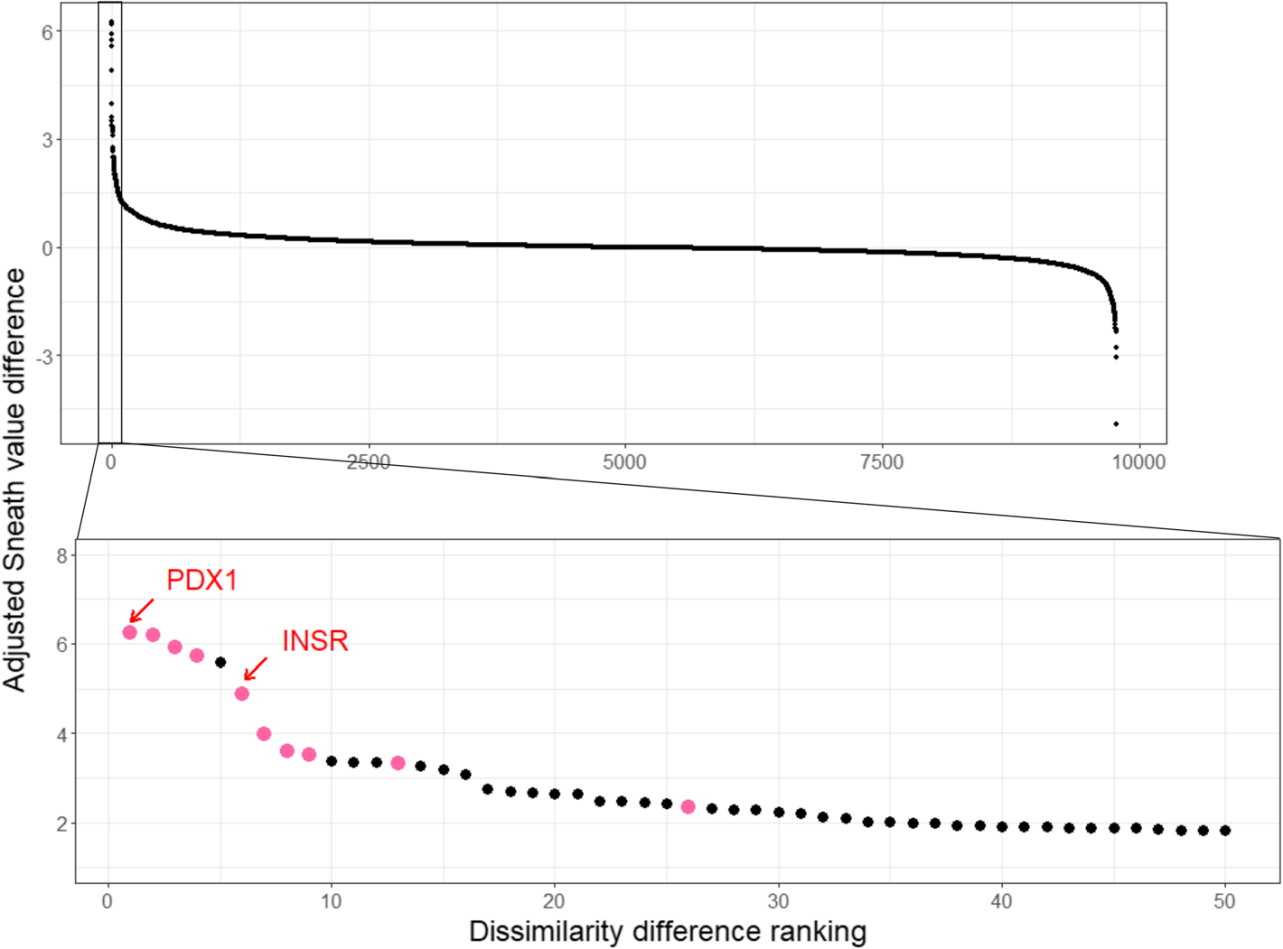
Dissimilarity difference ranking for 9771 sand rat proteins against the difference in adjusted Sneath value compared to the murid homologue. The top 61 ranked proteins are enlarged in the bottom plot with proteins PDX1 (rank 1) and INSR (rank 6) marked with arrows. Amongst these proteins, those encoded by genes in the extreme GC-rich region are shown in pink

A small fraction of proteins lies far from the x = y axis indicating differences in Sneath values. To identify those proteins that have ‘unusually high’ sequence divergence in gerbils compared to mice and rats, we first calculated the difference between adjusted Sneath values for sand rat and their corresponding murid protein average values and then ranked these ‘dissimilarity differences’ from largest to smallest. High ranking proteins are abnormally divergent in the sand rat compared to their mouse and rat homologues, while low ranking proteins are divergent in mouse and rat compared to the sand rat. Second, by comparing rank order with dissimilarity difference, we looked for discontinuities in the distribution that could be used to identify a set of ‘unusually’ divergent proteins (Fig. 2). Most proteins have a dissimilarity difference close to 0, while a small number of proteins differ drastically between the sand rat and murid lineage.

Discontinuities in the graph suggest there are 50 ‘aberrantly divergent’ protein coding genes in sand rat with difference values above 1.80, of which 16 are exceptionally unusual with Sneath dissimilarity differences above 3 (Additional file 2). In contrast, murids have 18 aberrantly divergent protein coding genes with difference values above 1.80, with just 2 highly unusual genes showing a difference value above 3 (Fig. 2; Additional file 1: Fig. S2).

To further test if these results are driven by unusual evolution in gerbils, and not slow evolution in mouse and rat, we also compared Sneath values for sand rat proteins to the average Sneath values of their homologues across a wider clade encompassing kangaroo rat (*Dipodomys ordii*), jerboa (*Jaculus jaculus*), blind mole rat (*Nannospalax galili)*, mouse and rat. This analysis did not change the overall picture, identifying 67 sand rat proteins (dissimilarity difference > 1.80) including 17 exceptionally unusual (dissimilarity difference > 3) (Additional file 1: Fig. S3).

To test whether the results of this analysis are applicable to other gerbil species, we compared Sneath values of 10,069 Mongolian jird genes to their murid orthologues, finding 41 aberrantly divergent genes in the Mongolian jird (Additional file 2). This number is lower than sand rat partly due to incompleteness of the Mongolian jird genome assembly. Average gerbil Sneath values were also compared to average murid Sneath values (Additional file 1: Fig. S4).

### Many divergent gerbil proteins are associated with high GC

It has been shown previously that gerbil genomes have islands of GC bias, and for the *Pdx1* gene (located in the island of most extreme GC) this is associated with radical protein sequence divergence [3, 5]. To test whether excess mutational change and/or GC bias has driven unusual protein sequence divergence in other gerbil genes, we focused on the 50 genes with aberrantly high relative sequence divergence in sand rats (highest adjusted Sneath value).

**Fig. 3:**
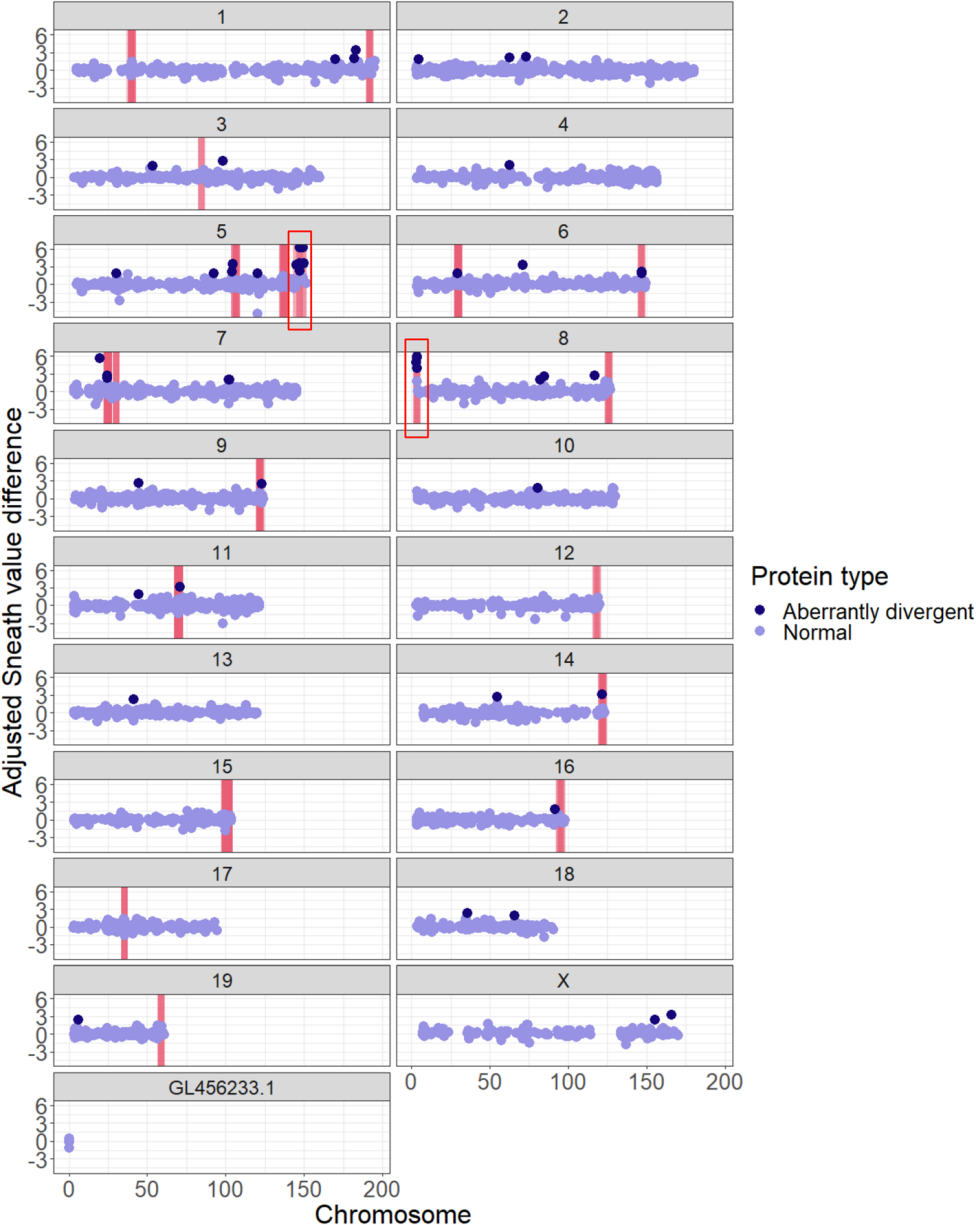
Aberrantly divergent sand rat proteins are frequently encoded by genes in GC-rich islands. Each panel shows one mouse chromosome (scale in Mb) to which the locations of sand rat orthologues are mapped. All analyzed sand rat genes are displayed as dots plotted according to the midpoint position of their corresponding mouse orthologue. The position of each dot on the y-axis shows the difference in adjusted Sneath value between the sand rat and mouse orthologues. Pink lines indicate locations of GC-rich regions identified previously [3, 5]. ‘Clusters’ of aberrantly divergent proteins with more than two proteins mapped to regions less than 1 Mb apart are marked with open red boxes

First, we asked how many of the aberrantly divergent proteins are encoded by genes located in the most extreme GC-rich region of the sand rat genome (the set of genes including *Pdx1*) [3, 5]. Our dataset of 9771 orthologous genes includes 14 genes from the extreme GC-rich region in the sand rat genome, out of which 10 are present in the set of 50 most aberrantly divergent protein sequences (Fig. 3). Extreme GC-rich genes are significantly enriched in the set of aberrantly divergent proteins (Fisher’s Exact Test; *p*-value = 4.7e-21). These 10 genes are *Pdx1*, *Medag*, *Pex11g*, *Tex45*, *Insr*, *Trappc5*, *Pan3*, *B3glct*, *Pdap1*, and *Cdx2*.

We extended our analysis beyond the extreme GC-rich genomic region and tested if other aberrantly divergent genes are associated with other less extreme high GC regions identified previously in the sand rat genome [3, 5] (Additional file 1: Table S1). In total, we found 19 of the 50 most aberrantly divergent proteins are encoded by genes in known high GC regions (including the 10 noted above), compared to 277 out of 9721 non-divergent genes in these regions. This is a significant enrichment of aberrantly divergent proteins encoded within the known GC-rich islands of the sand rat genome (Fisher’s Exact Test; *p*-value = 1.0e-16). Even when we removed the genes located in the extreme high GC region from this analysis, the enrichment is significant (9/40 of divergent proteins in high GC regions, compared to 273/9717 non-divergent proteins; Fisher’s Exact Test *p*-value = 1.5e-06). Using high GC region coordinates previously reported for the Mongolian jird [5], we also found a similar enrichment of aberrantly divergent proteins in GC regions (Fisher’s Exact Test; *p*-value = 9.6e-04) (Additional file 1: Table S2 and Fig. S5).

The above tests focus on the chromosomal location of each gene and the nucleotide compositional processes affecting different genomic regions. We also analyzed genes individually to enable a higher resolution analysis of the patterns of nucleotide substitution underpinning protein divergence. Amino acid substitutions, which occurred to an extreme extent in the aberrantly divergent proteins, are caused by non-synonymous substitutions. However, here we focus on synonymous substitution rates (dS) to enable us to detect the presence of underlying mutational processes, independent of selection on amino acid changes. Specifically, we tested whether aberrantly divergent gerbil proteins are encoded by genes that have experienced GC bias (an excess of weak to strong mutations) and/or an excess of mutational change in all categories. We calculated dS values for all categories of nucleotide changes: weak-to-strong (dS_ws_ = rate of A or T being altered to C or G), strong-to-weak (dS_sw_ = rate of G/C to A/T), weak-to-weak (dS_ww_) and strong-to-strong (dS_ss_). In this paper, we defined dS ‘outliers’ as genes with dS values more than 2.5 times the average dS for that category [5].

Out of the 50 genes encoding aberrantly divergent proteins, 52% are dS_ws_ outliers (26/50) compared to only 4% of the remaining genes (358/9721), showing that dS_ws_ outlier genes are significantly enriched for aberrantly divergent proteins (Fisher’s Exact Test; *p*-value = 6.3e-24). In addition, 19 out of 26 dS_ws_ outliers encoding aberrantly divergent proteins are in high GC regions highlighted in Fig. 3. We also find that dS_sw_, dS_ww_, or dS_ss_ outliers are significantly enriched for aberrantly divergent proteins (dS_sw_ *p*-value = 4.4e-30; dS_ww_ *p*-value = 4.4e-19; dS_ss_ *p*-value = 8.8e-25) (Additional file 1: Table S3-S5). These results indicate that many of the aberrantly divergent gerbil proteins are encoded by genes that have experienced unusually high levels of mutational change, even at synonymous sites, and especially high levels of weak-to-strong mutations.

**Fig. 4:**
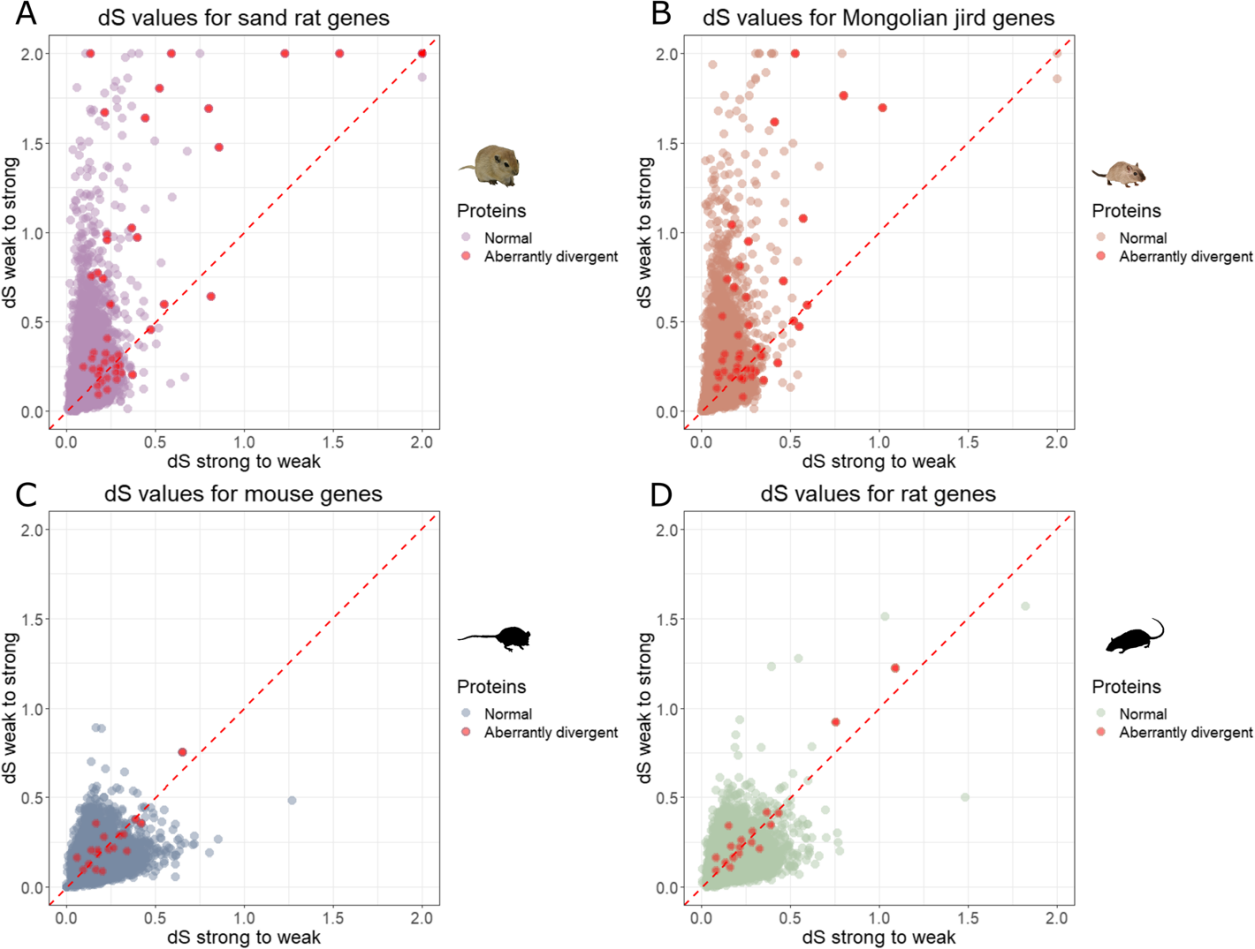
Mutational patterns at synonymous sites for orthologous genes from four rodent species. Genes encoding aberrantly divergent proteins are highlighted with red dots. **a** Graph comparing weak-to-strong (dS_ws_) and strong-to-weak (dS_sw_) synonymous mutation rates for sand rat genes. The ‘chimney’ shape indicates that many sand rat genes have undergone GC biased nucleotide changes; many of the aberrantly divergent proteins are in this category. Eighteen sand rat genes (including 12 aberrantly divergent genes) with dS_ws_ and/or dS_sw_ values above 2 have been artificially converted to dS_ws_ = 2 and/or dS_sw_ = 2 to give a more comprehensive view. **b** Graph comparing weak-to-strong (dS_ws_) and strong-to-weak (dS_sw_) synonymous mutation rates for Mongolian jird genes. The ‘chimney’ shape indicates GC bias; most aberrantly divergent proteins are in this category. Ten Mongolian jird genes (including two aberrantly divergent genes) with dS_ws_ and/or dS_sw_ values larger than 2 have been artificially converted to dS_ws_ = 2 and/or dS_sw_ = 2. **c** Graph comparing dS_ws_ and dS_sw_ for mouse genes. **d** Graph comparing dS_ws_ and dS_sw_ for rat genes. Photographs from J.F. Mulley and Pixabay with permission

To test if these elevated mutational effects are associated with GC bias (excess of change towards G or C nucleotides), we measured the ratio of dS_ws_ to dS_sw_ (high dS_ws_ causes GC content to increase, high dS_sw_ causes AT content to increase). Plotting dS_ws_ for each gene against dS_sw_ shows a large number of genes in gerbils with GC bias, as previously reported (sand rat dS_ws_/dS_sw_ = mean 1.81; Mongolian jird dS_ws_/dS_sw_ = mean 1.99; Fig. 4a and b) [5]. The same effect is not seen in murid rodents (mouse dS_ws_/dS_sw_ = mean 1.20; rat dS_ws_/dS_sw_ = mean 1.24; Fig. 4c and d). Most of the aberrantly divergent proteins (37 out of 50 in sand rat, 28 out of 41 in Mongolian jird) in gerbils lie above the x = y line (sand rat aberrantly divergent genes dS_ws_/dS_sw_ = mean 2.19; Mongolian jird aberrantly divergent genes dS_ws_/dS_sw_ = mean 1.97; Fig. 4a and b). For aberrantly divergent sand rat genes not located in GC-rich regions, average dS_ws_/dS_sw_ = 2.23, showing that aberrantly divergent genes are disproportionately affected by GC bias regardless of whether they are in GC-rich regions. These results imply that high mutation accumulation and GC bias are major factors contributing to the evolution of divergent gerbil proteins.

### Some aberrantly divergent gerbil proteins show signs of positive selection

An alternative hypothesis to explain the divergence observed in the 50 gerbil proteins is that these have been under strong positive selection in the gerbil lineage relative to the other rodent species. To test this hypothesis, we performed branch tests using the PAML program [35]. Of the 50 aberrantly divergent sand rat proteins, we identified 21 predicted to have a higher dN/dS in the gerbil lineage compared to other rodent lineages (Additional file 1: Fig. S6, S7). However, only 3 had dN/dS > 1 indicative of strong positive selection: TEX37 (Testis Expressed 37), SYCP2L (Synaptonemal Complex Protein 2 Like) and IL15 (Interleukin 15) (Additional file 2). None of the three have dS_ws_ or dS_sw_ values above 0.5 and all have dS_ws_/dS_sw_ < 1, showing that these were not affected by GC bias (Additional file 1: Fig. S7A).

We then used a branch-site test to identify sand rat proteins that show evidence of positive selection at specific residues [36]. We used an implementation of this test that incorporates codon substitution rate variation and thereby controls for variation in the synonymous substitution rate caused by factors such as GC-biased gene conversion [37]. We identified 13 aberrantly divergent sand rat proteins with evidence of positive selection at specific residues (Additional file 1: Fig. S7B, S8). These include one of the positively selected proteins identified by the branch test model, TEX37, and six of the 26 proteins with an outlying dS_ws_. These results show that positive selection has contributed to the evolution of some aberrantly divergent gerbil proteins but is not the major force leading to most of the observed extreme amino acid differences.

### Phenotypic implications of aberrantly divergent gerbil genes

We asked if the evolution of aberrantly divergent proteins, driven primarily by elevated mutational and gBGC processes, could have phenotypic consequences for gerbils. Specifically, we wished to test if the evolution of divergent gerbil genes could be associated with the type 2 diabetes-prone phenotype observed in sand rats and Mongolian jirds.

First, we performed Gene Ontology (GO) analyses to test for over-representation of biological functions or molecular pathways in the set of aberrantly divergent genes, and as a control we also analyzed the initial set of 9771 genes. We found significant enrichment of several biological processes, molecular functions, and cellular components in the dataset of 9771 sand rat genes when compared to the mouse genome (Panther; Fisher’s Exact Test, q-value < 0.05), but overrepresentation was modest with maximum fold-change 2.01. Other functions showed underrepresentation including sensory perception of smell (GO:0007608; 20-fold underrepresentation, q-value = 5.44e-137), likely a result of only using 1-to-1 orthologues causing bias against dynamic gene families with extensive gene duplication and loss. However, when using Metascape to test for enrichment in the dataset of 9771 sand rat genes compared to the mouse genome, we did not detect significant enrichment or underrepresentation of any GO terms. We did not find significant overrepresentation or underrepresentation for biological processes, functions or cellular components in the aberrantly divergent proteins when compared against the dataset of 9771 sand rat genes (Panther and Metascape; q-value < 0.05). We interpret these results to mean that the aberrantly divergent proteins that evolved in gerbils are associated with a diversity of biological functions, not only dietary metabolism.

**Fig. 5:**
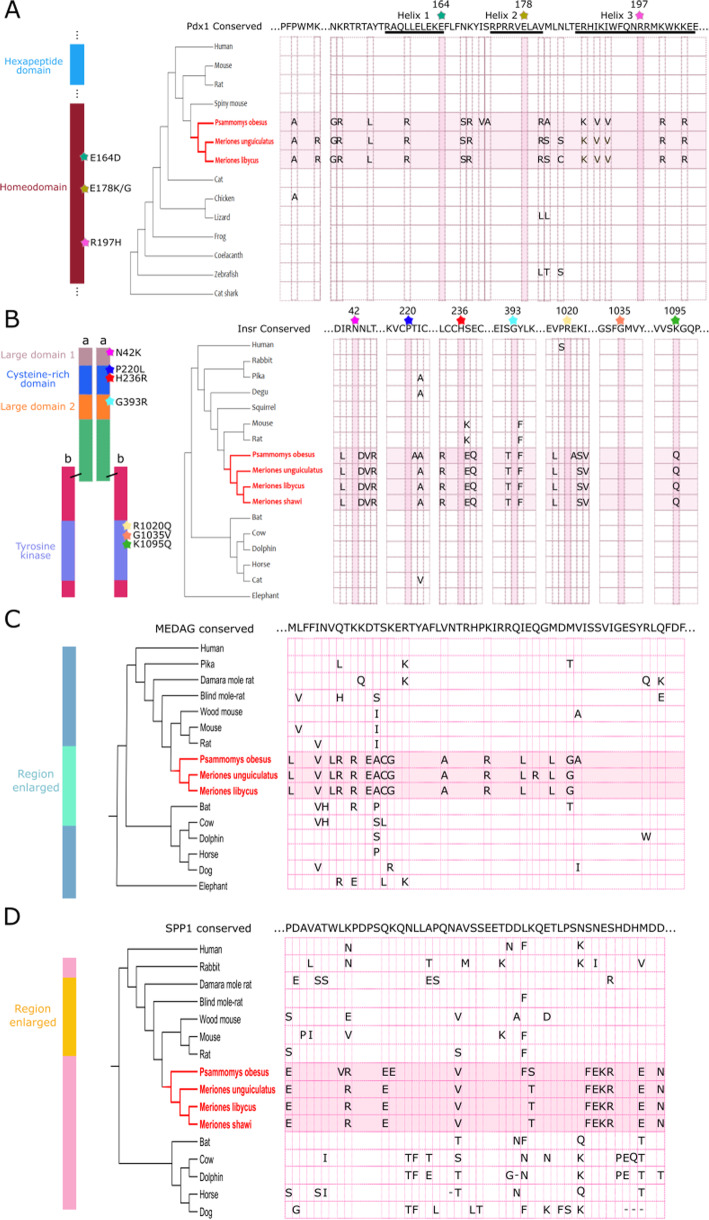
Alignment of key functional domains in PDX1, INSR, MEDAG, and SPP1 proteins. **a** Alignment of the conserved PDX1 hexapeptide and homeodomain sequence from representative vertebrates. Gerbil species shown in red; sites where amino acid substitutions are associated with T2D in humans are marked with a star. **b** Alignment of regions for four domains in the INSR protein. Due to sequence divergence across vertebrates, only sequences from mammals are shown. Gerbil species are shown in red; sites where amino acid substitutions are associated with T2D in humans are marked with a star. **c** Alignment of a representative region of the MEDAG protein. Gerbil species shown in red. **d** Alignment of a representative region of the SPP1 protein. Gerbil species are shown in red

To search for possible associations between aberrant protein divergence and propensity to type 2 diabetes, we took a candidate gene approach. Specifically, we asked if any of the proteins showing extreme relative divergence in gerbils have been linked to dietary metabolism in other species. We note that amongst the top 10 most aberrantly divergent sand rat proteins, four are clearly associated with lipid or carbohydrate metabolism. The genes encoding these are all located in the extreme GC-rich region of sand rat genome (Table 1). These four proteins are the previously discussed PDX1 transcription factor, plus MEDAG (Mesenteric Estrogen Dependent Adipogenesis), INSR (Insulin Receptor) and SPP1 (Secreted Phosphoprotein 1 or osteopontin). In Fig. 5, we show protein sequence alignments of these four proteins to highlight some of the highly unusual amino acid changes observed in sand rat and related gerbil species, compared to other mammals. In the PDX1 protein, there is a high degree of conservation across vertebrates of the hexapeptide domain (a cofactor binding domain) and the homeodomain (DNA-binding and sequence recognition domain), but extreme divergence in three gerbil species (Fig. 5a) [3, 5]. For INSR, a strong candidate for association with metabolic function, we find many amino acid changes unique to the gerbil lineage throughout the protein, although nearly all key amino acid sites previously associated with T2D in humans remained conserved (Fig. 5b). For MEDAG and SPP1, we also observe a number of gerbil-specific amino acid residues in mammal-conserved regions (Fig. 5c and d).

**Table 1:** A list of the sand rat genes that encode the most aberrantly divergent sand rat proteins ordered by ranking, whether these genes are in GC-rich regions, dS_ws_ value and a short description of the function of their encoded protein

Rank	Gene	GC-rich region	Sand rat dS_**ws**_ value	Encoded protein
1	*Pdx1*	Yes	20.09	Pancreatic duodenal homeobox. Transcription factor involved in pancreas development and β-cell function.
2	*Medag*	Yes	3.09	Mesenteric estrogen dependent adipogenesis. Involved in adipocyte differentiation and glucose uptake.
3	*Pex11g*	Yes	4.58	Peroxisomal membrane protein. Involved in growth and division of peroxisomes (organelles partaking in lipid biosynthesis).
4	*Tex45*	Yes	1.48	Testis-expressed protein. Understudied gene expressed in testis.
5	*Cblc*	No	0.60	E3 ubiquitin-protein ligase. Involved in protein degradation; down-regulates receptor-tyrosine kinase (RTK) signaling.
6	*Insr*	Yes	7.08	Insulin receptor. RTK receptor involved in insulin signaling and stimulation of glucose uptake.
7	*Trappc5*	Yes	34.40	Trafficking protein particle complex subunit. Implicated in vesicular transport from ER to Golgi.
8	*Pan3*	Yes	10.66	Subunit of PAN2-PAN3 deadenylase complex. Trims poly(A) tail prior to degradation.
9	*B3glct*	Yes	2.29	Beta 3-glucosyltransferase. Transfers glucose onto fucose on TSR repeat proteins.
10	*Spp1*	No	0.22	Secreted phosphoprotein 1 or osteopontin. Implicated in bone mineralization, immune system, and diet-induced insulin resistance.

## Discussion

When unusual or divergent proteins are observed in some species and not others, this is generally thought to result from natural selection acting to adjust or optimize a protein for a new or modified function. The contributions of mutation rate variation and recombination frequency differences between species are easily overlooked. However, there is growing evidence that these genomic and chromosomal level processes can have dramatic effects on the way that genes and proteins evolve. In this study, we focus on two gerbil species that have unusual GC-rich regions scattered through their genome, where recombination-related processes are causing accumulation of G and C nucleotides [5]. Previous work showed that GC-biased substitutions in one of these regions caused deleterious amino acid changes in a sand rat protein, PDX1 [4]. Here we analyse the predicted proteomes of two gerbil species to identify proteins that are aberrantly divergent compared to other rodent species. We then test whether these proteins have become divergent because of association with GC-rich genomic regions and GC bias, rather than because of positive selection. In addition, we ask whether any of the aberrant proteins could be linked to physiological abnormalities observed in some gerbils.

Aberrantly divergent gerbil proteins have an extremely high number of amino acid changes at sites that are conserved in other rodent lineages, or indeed conserved across the vertebrates. The conservation of amino acid sequence is indicative of purifying (negative) selection acting to prevent change, and thus it is striking to see such major changes in conserved proteins. One possible hypothesis to explain the aberrantly divergent proteins is that strong positive selection and adaptive changes occurred in the gerbil lineage. The overall distribution of dN/dS across both gerbil genomes and murid genomes is similar, although both gerbil species exhibit a stronger right-skew, indicating presence of more genes with high dN/dS (Additional file 1: Fig. S6). It is unlikely that this is an artefact of poor genome sequence or assembly quality as the sand rat genome has a high scaffold N50 (8.8 Mb) and coding sequences of several divergent sand rat genes were verified by transcriptome sequencing [3]. Furthermore, sequence alignment between gerbil species reveals similar changes in some proteins (Fig. 5). Effective population size (Ne) can also affect dN/dS distribution, as natural selection is less efficient in small populations [7]. We do not have sufficient information about demographic history to evaluate its contribution to dN/dS distribution..

Using the branch test, we detect signals of strong positive selection in only 3 out of 50 aberrantly divergent proteins (Additional file 2). These are TEX37 (Testis Expressed 37), SYCP2L (Synaptonemal Complex Protein 2 Like) and IL15 (Interleukin 15); they are not associated with GC bias nor located in GC-rich regions. TEX37 is predominantly expressed in the testis [38] while SYCP2L is mainly expressed in the ovary [39]. In addition, gene association studies report a correlation between *SYCP2L* variants and lipid metabolism, although the mechanism remains unknown [40, 41]. IL15 is a proinflammatory cytokine that activates T-cells and aberrant activity has been linked to destruction of pancreatic beta-cells in type 1 diabetes (T1D) [42, 43]. However, autoimmune destruction of β-cells has not been postulated as a cause of metabolic dysfunction in gerbils. In general, reproductive proteins and immunity-related proteins are under positive selection in many species [44, 45].

A second possibility is that these aberrant proteins have lost function and are randomly accumulating amino acid changes. This is again unlikely given that we do not observe early stop codons or frameshifts in the coding sequences of these genes. A third hypothesis is that genomic processes have inadvertently caused unusual sequence changes to become fixed in populations, even if slightly deleterious at the time of origination. For example, GC-biased gene conversion (gBGC) can convert heterozygous sites to homozygous sites, and overall gBGC promotes the fixation of neutral and slightly deleterious weak-to-strong substitutions [8, 46]. This hypothesis predicts that aberrantly divergent proteins would be enriched for genes located in GC rich genomic regions, as these are localized regions where gBGC has occurred at high rates. Consistent with this prediction, we find a significant enrichment of aberrantly divergent genes mapping to previously described high GC regions in the gerbil genome [3, 5]. Also consistent with this hypothesis, aberrant gerbil genes are enriched for dS_ws_ outliers and show strong GC bias in gerbils (sand rat average dS_ws_/dS_sw_ = 2.19) compared to murids (mouse average dS_ws_/dS_sw_ = 1.20). In addition, aberrant gerbil genes are enriched for dS_sw_, dS_ww_, and dS_ss_ outliers, suggesting that mutational processes aside from gBGC also contribute to excess nucleotide changes affecting these genes. For example, the process of recombination is mutagenic and cytosine (strong) to thymine (weak) changes occur at high rates near recombination sites [47]. In general, point mutations are AT-biased, associated with high rates of spontaneous deamination leading to conversion of cytosine (strong) to thymine (weak) [48].

We argue that many (but not all) of the aberrantly divergent gerbil genes are likely to have arisen through unusually high levels of nucleotide substitution, including especially high levels of weak-to-strong substitutions resulting in GC bias. Given the degree of amino acid change observed in these gerbil proteins, the high rates of nucleotide substitution have likely resulted in fixation of slightly deleterious mutations in these proteins, with natural selection only successfully removing individuals with the most severely deleterious amino acid changes. We suggest that gerbil evolution has witnessed a tug-of-war between localized GC bias in certain genomic regions causing fixation of deleterious mutations and natural selection preserving essential genes trapped in these ‘quagmires’ of GC bias.

The two gerbil species focused on in this study have also been reported to become obese and exhibit metabolic abnormalities when maintained on a standard laboratory diet or a high fat diet, although not in the wild [23, 25, 49, 50]. For example, sand rat islet cells show more pancreatic β-cell damage compared to rat islets when exposed to high glucose concentrations, and even healthy sand rats on plant-based diets do not have a strong response to human insulin [51, 52]. Could these metabolic disorders be related to the accumulation of deleterious mutations in gerbils, driven by excessive biased gene conversion? We do not have direct evidence linking specific genetic changes with phenotype, but we argue there is a plausible connection. We highlight four aberrantly divergent gerbil proteins for which the human and mouse orthologues have been associated with dietary metabolism: PDX1, INSR, MEDAG and SPP1. In two cases, the human orthologues have been associated directly with type 2 diabetes or other metabolic diseases, and these associations can be tracked down to single amino acid substitutions or other small mutations. In gerbils we see dramatic amino acid change at multiple conserved sites. In the other two cases, functional studies in mouse or human indicate key roles in adipose tissue.

PDX1 is a homeodomain transcription factor essential for vertebrate pancreatic development and normal β-cell function [15–17]. PDX1 is highly conserved across vertebrates, and *PDX1* mutations have been linked to metabolic disease in humans. The homeodomain is the most highly conserved protein domain in PDX1 and point mutations in this region severely compromise PDX1 function. For example, an individual born with pancreas agenesis was reported to carry a E164D substitution on one allele and a E178K substitution on the other [19]. In addition, the mutation R197H has > 50% decreased binding affinity with the human insulin promoter sequence and is associated with type 2 diabetes [20]. These conditions in humans are associated with single amino acid changes to the homeodomain, yet by contrast the sand rat PDX1 homeodomain has a total of 15 amino acid differences from the conserved sequence (Fig. 5a). It is the most aberrantly divergent protein out of 9771 analyzed proteins in the present study, and it is likely that the combination of a large number of substitutions would radically compromise protein function.

INSR, the insulin receptor, is the cell surface receptor for insulin and IGF peptides and initiates the insulin signaling pathway in vertebrates [53]. In sand rat, the INSR protein is the 6th most aberrantly divergent protein and carries many substitutions at otherwise conserved sites. Recent work has uncovered adaptive change in the INSR protein of Mexican cavefish, notably a P211L substitution mutation (equivalent to P220L in human INSR) fixed by positive selection [54]. This mutation reduces binding capability to the insulin peptide and results in insulin resistance in the cavefish, which paradoxically may be a trait beneficial to survival in a cave environment lacking a stable food supply [54]. In humans, deleterious mutations affecting INSR function have been reported, with over 30 different point mutations described [31]. In gerbils, such as sand rat, we find many unusual changes that generally do not match the individual mutations found in cavefish or associated with metabolic conditions, apart from point mutation K1095Q. The large number of changes, and the association with GC bias, are not consistent with adaptive change.

For the two other aberrant proteins putatively associated with dietary metabolism, MEDAG and SPP1, we cannot make comparisons to variants associated with human phenotypes. However, the known functions of these proteins suggest plausible association with dietary metabolism. MEDAG is a well-conserved pro-inflammatory protein that promotes adipocyte differentiation and regulates adipocyte glucose uptake [55]. Islet expression of *MEDAG* is different between diabetic and healthy individuals [56], and copy number variation in the MEDAG loci is associated with obesity [57]. SPP1, also known as osteopontin, is a multifunctional protein involved in biomineralization and bone remodeling. SPP1 also has roles in adipose tissue and contributes to adipose tissue inflammation and insulin resistance [58]. Mice fed on a high fat diet, or with genetic predisposition to obesity, show enormous elevation of SPP1 expression levels [59], while blocking SPP1 function by antibodies or genetic mutation improves insulin sensitivity [60, 61]. In humans, SPP1 expression is also elevated in adipose tissue of obese individuals [59, 62]. We speculate, therefore, that the amino acid changes in these gerbil proteins likely cause changes in protein function and could contribute to the unusual dietary physiology of gerbils.

The large number of amino acid changes in these proteins is not consistent with adaptive change, while the association with GC bias and greatly elevated synonymous substitution rates is indicative of high rates of gBGC causing some deleterious mutations to be fixed. Despite the unusual finding of deleterious mutations accumulating in key genes, we expect that the most severely detrimental amino acid changes in PDX1, INSR, MEDAG and SPP1 (and other proteins) will have been removed by natural selection. We also suggest that amino acid changes that may be deleterious at the time of origin might be partially compensated for by adaptive change selected for in other genes. For example, mutations adversely affecting dietary physiology might be partially compensated by selection affecting traits such as dietary choice, satiation detection or habitat use.

## Conclusions

In this paper, we show there are more than twice the number of aberrantly divergent proteins in gerbils compared to mice and rats, and that many of the genes that encode these aberrant proteins are associated with GC bias or previously reported GC-rich genomic regions [3, 5]. We propose that a tug-of-war between GC bias and natural selection has been taking place during gerbil evolution, leaving behind an excess of deleterious mutations in several aberrant gerbil genes, some of which may be partly responsible for the abnormal metabolic phenotypes observed in gerbil species.

## Methods

### Curation and alignment of 1-to-1 orthologues

We identified and aligned groups of orthologous sequences following a previously described approach [5]. Briefly, we obtained predicted gene sequences for 12 rodents and human from Ensembl (release 95, accession IDs of genome annotations in Additional file 1: Table S6 and coding sequence and protein IDs in Additional file 2), retrieved the longest transcript for each gene and identified groups of orthologous transcripts between these species using Orthofinder version 2.2.7 [63, 64] applying default parameters, the diamond aligner version 0.9.21 [65] and the species tree topology shown in Fig. 1a. From these, we identified groups of orthologous genes that have a single sequence in (a) both murids (mouse and rat) and the outgroup species (human), (b) at least one gerbil (sand rat and/or Mongolian jird) but not more than one sequence in either species, and (c) all but one or two of the remaining species, in which case we removed those species from the analysis. This approach was chosen to maximize the number of genes analyzed despite assembly errors and occasional gene duplications or deletions in rodent genomes.

Protein and nucleotide sequences of each orthologous gene set were aligned using MACSE v2.03 with default parameters and recoded with the MACSE exportAlignment function: options ‘-codonForInternalStop NNN -codonForExternalFS --- -codonForInternalFS ---’ [66]. We removed low similarity regions using the default parameters of HmmCleaner.pl version 0.180750 [67]. Additional filtering was performed with MACSE reportMaskAA2NT function, with parameters ‘-min_NT_to_keep_seq 30 -mask_AA $ -min_seq_to_keep_site 4 -min_percent_NT_at_ends 0.3 -dist_isolate_AA 3 -min_homology_to_keep_seq 0.3 -min_internal_homology_to_keep_seq 0.5’. We also filtered each alignment by removing any sequence for which gaps represented more than 70% of the non-gap length of the longest gene in the alignment. Since this removes species from alignments, we again applied the species filter used above, requiring both murids and human to be single copy, at least one gerbil to be single copy, and all but one or two other species to be single copy. DNA alignments for the resulting 10,554 orthologue datasets can be found in Additional file 3.

### dN and dS calculation

For each orthologue dataset, the tree in Fig. 1a was trimmed to only include the species in that alignment. Branch lengths for each alignment were optimized using the BppML subprogram version 2.3.1 of BppSuite [68], the YN98 (F3X4) model [69] and parameters available online [70]. Synonymous and nonsynonymous substitution rates (dS and dN, respectively) were estimated for each mutational category (weak-to-strong, strong-to-weak, weak-to-weak, and strong-to-strong) using the parameter ‘map.type = Combination (reg1 = dNdS, reg2 = SW)’ of the BppML subprogram MapNH version 1.1.1 [71]. Branch lengths were summed from the Muridae node to the tree tip to retrieve rate measurements for mouse, rat, sand rat and Mongolian jird.

To identify genes under positive selection, we calculated the dN/dS ratio of each gene using the codeml function in the PAML package [35]. As input, we used the DNA alignments for each 1-to-1 orthologue from the previous analysis (Additional file 3) and the phylogenetic tree shown in Fig. 1a. For each orthologue, we performed a likelihood ratio test (LRT) between a null model (NSsites = 0, model = 0) and a two-ratio branch model (NSsites = 0, model = 2) with the gerbil species and the ancestral branch of gerbils marked as the foreground branch; False Discovery Rate correction for multiple-testing used the qvalue library in R [72]. The dN/dS obtained from the two-ratio branch model was used if LRT results rejected the null model. We used the skewness function in the R package moments [73] to calculate skewness of genome-wide dN/dS distribution in each species. In addition, we performed branch-site analysis for each orthologue using the Godon program [37], first optimizing the branch lengths with the M0 model, then running the branch-site model with codon gamma rate variation (−-ncat-codon-rate 4) with the gerbil species and the ancestral branch of gerbils marked as the foreground branch [37]. We computed the resulting *p*-values with likelihood ratio tests (LRT) between the null and alternative models performed by the program. The resulting *p*-value distribution had a strong excess of cases where *p* > 0.99 (Additional file 1: Fig. S9), precluding the direct use of false discovery rate methods for multiple testing correction. To correct for multiple testing, we therefore removed all tests where *p* > 0.99 and corrected the remaining *p*-values using the robust FDR estimation method for 1-sided tests [74].

### Protein dissimilarity calculation and ranking

From the protein alignments, we removed any sites containing a stop codon, an unknown residue, or a missing residue in one or more species. We then identified all sites which had the same amino acid in three outgroup species. For most proteins we used human (*Homo sapiens*), guinea pig (*Cavia porcellus*) and squirrel (*Ictidomys tridecemlineatus*) as the outgroup species. However, for seven alignments we used human, degu (*Octodon degus*) and chinchilla (*Chinchilla lanigera*) as they lacked the guinea pig and squirrel sequence; for 376 alignments we used human, guinea pig and jaculus (*Jaculus jaculus*) as they lacked the squirrel sequence; and for 270 alignments we used the human, squirrel and degu sequence as they lacked the guinea pig sequence. Sites with the same amino acid in all three outgroups were defined as ‘conserved positions’; only these sites were used to calculate protein dissimilarity. Proteins with < 50 residues meeting this criterion were excluded, reducing the dataset to 9771 genes for sand rat and 10,069 for Mongolian jird.

To calculate a Sneath score [33], each site which differed from a ‘conserved position’ was assigned a value dictated by the Sneath Index, and values summed to produce the Sneath value for the protein in that species. Epstein’s Coefficient assigns different values to each amino acid difference; these were summed in the same way [34]. Scores were divided by the length of the parsed alignment to produce adjusted protein dissimilarity values.

### GC bias analysis and correlation with high GC peaks

To measure GC bias, synonymous substitution rates for weak to strong changes (dS_ws_) were divided by dS for strong to weak changes (dS_sw_), estimated using the species tree and calculated from the murid-gerbil node to the terminal branch tips. Genes with dS_ws_/dS_sw_ > > 1 are under strong GC bias, and genes with dS_ws_/dS_sw_ < < 1 are under strong AT bias. To test for correlation with position, 10,000 random samples of 50 sand rat genes from the 9771 proteins dataset or 50 Mongolian jird genes from the 10,069 orthologues dataset were selected and chromosomal location in mouse recorded; samplings were compared to locations for the 50 genes with highest adjusted Sneath value. To test for correlation with high GC peaks or islands in the sand rat and Mongolian jird genomes, we used peaks defined previously using sliding window analysis of dS_ws_ [5] (coordinates in Additional file 1: Table S1).

### Analysis of protein functions

To test if ‘aberrantly divergent’ sand rat proteins are enriched for biological functions, we tested for statistical overrepresentation using PANTHER and Fisher’s Exact test with calculation of False Discovery Rate as the correction method [75, 76]. PANTHER identified mouse orthologue IDs for 9671 of the 9771 proteins of interest (including 49 out of 50 aberrantly divergent proteins). We analyzed the same dataset using Metascape [77] which identified mouse orthologue IDs for 9752 out of the 9771 sand rat proteins of interest (including 50 out of 50 aberrantly divergent proteins).

## Supplementary information

**Additional file 1: Figures S1-S8.** Tables S1-S6. Patterns of protein divergence calculated using Epstein’s Coefficient (**Fig. S1**). Protein dissimilarity ranking for aberrantly divergent murid proteins, dissimilarity ranking calculated using the average of five murid species, and dissimilarity ranking calculated using the average of two gerbil species (**Fig. S2-S4**). High GC regions used for analysis (**Table S1**). Correlation between aberrantly divergent genes, high GC regions and dS outliers (**Table S2-S5**). Location of highly aberrant Mongolian jird genes (**Fig. S5**). Genome-wide distribution of dN/dS (**Fig. S6**). Relation between aberrantly divergent sand rat proteins and evidence of positive selection (**Fig. S7**). Distribution of *p*-values generated by Godon (**Fig. S8**). Accession IDs for genomes used in analysis (**Table S6**).

**Additional file 2 Supplementary Data.** Accession IDs for nucleotide sequences and protein sequences used in analysis. Raw Sneath values and Epstein values for all orthologues. The ranking of 9771 1-to-1 orthologues containing a sand rat gene calculated using the Sneath Index and Epstein’s Coefficient are provided in separate sheets. The ranking of 10,069 1-to-1 orthologues containing a Mongolian jird gene calculated using the Sneath Index and Epstein’s Coefficient are also provided.

**Additional file 3 Supplementary Data.** DNA alignments for 10,554 1-to-1 orthologues used in analysis. Each alignment file is named using its orthogroup ID.

## Data Availability

All data generated or analyzed during this study are included in this published article and its additional information files. Scripts used for analyses have been deposited on GitHub and archived in Zenodo [78].

## Abbreviations

GC: Guanine and cytosine
AT: Adenine and thymine
gBGC: GC-biased gene conversion
T2D: Type 2 diabetes
GO: Gene Ontology
RTK: Receptor-tyrosine kinase
