# Supplementary material for: Divergent genes in gerbils: prevalence, relation to GC-biased substitution, and phenotypic relevance
Source: BMC Evol Biol. 2020 Oct 19;20:134. doi: 10.1186/s12862-020-01696-3 (PMC7574485; doi:10.1186/s12862-020-01696-3)
Supplement: Supplementary file 1 — Additional file 1: Figures S1-S8. Tables S1-S6. Patterns of protein divergence calculated using Epstein’s Coefficient (Fig. S1). Protein dissimilarity ranking for aberrantly divergent murid proteins, dissimilarity ranking calculated using the average of five murid species, and dissimilarity ranking calculated using the average of two gerbil species (Fig. S2-S4). High GC regions used for analysis (Table S1). Correlation between aberrantly divergent genes, high GC regions and dS outliers (Table S2-S5). Location of highly aberrant Mongolian jird genes (Fig. S5). Genome-wide distribution of dN/dS (Fig. S6). Relation between aberrantly divergent sand rat proteins and evidence of positive selection (Fig. S7). Distribution of p-values generated by Godon (Fig. S8). Accession IDs for genomes used in analysis (Table S6). [file 12862_2020_1696_MOESM1_ESM.pdf]

Additional File 1 for:

Divergent genes in gerbils: prevalence, relation to GC-biased substitution, and phenotypic relevance

Yichen Dai, Rodrigo Pracana and Peter WH Holland *

Content:

Section 1: Protein dissimilarity calculated using Epstein's Coefficient

Section 2: Protein dissimilarity ranking

Section 3: Association between aberrantly divergent proteins, high GC islands, and selection

Section 4: Curation and alignment of 1-to-1 orthologues

Section 1: Protein dissimilarity calculated using Epstein's Coefficient

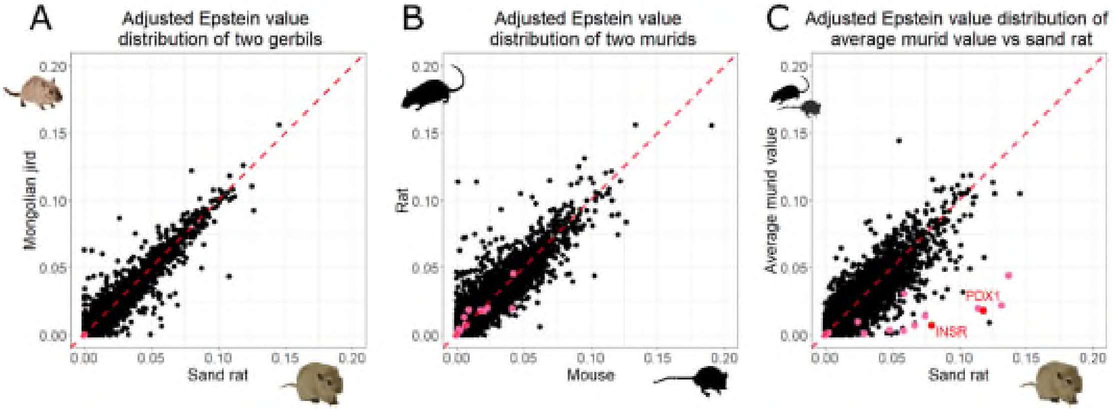

Fig. S1 Patterns of protein sequence divergence. Adjusted Epstein values orthologous proteins compared between (A) two gerbil species, (B) two murid species, (C) the average of two murid species and the sand rat. Each point represents one protein; proteins encoded by genes in the extreme GC-rich region of sand rat are shown in pink or (for PDX1 and INSR) in red. Only one protein encoded within the extreme GC-rich region is present in (A) due to missing genes in the Mongolian jird genome assembly.

Section 2: Protein dissimilarity ranking

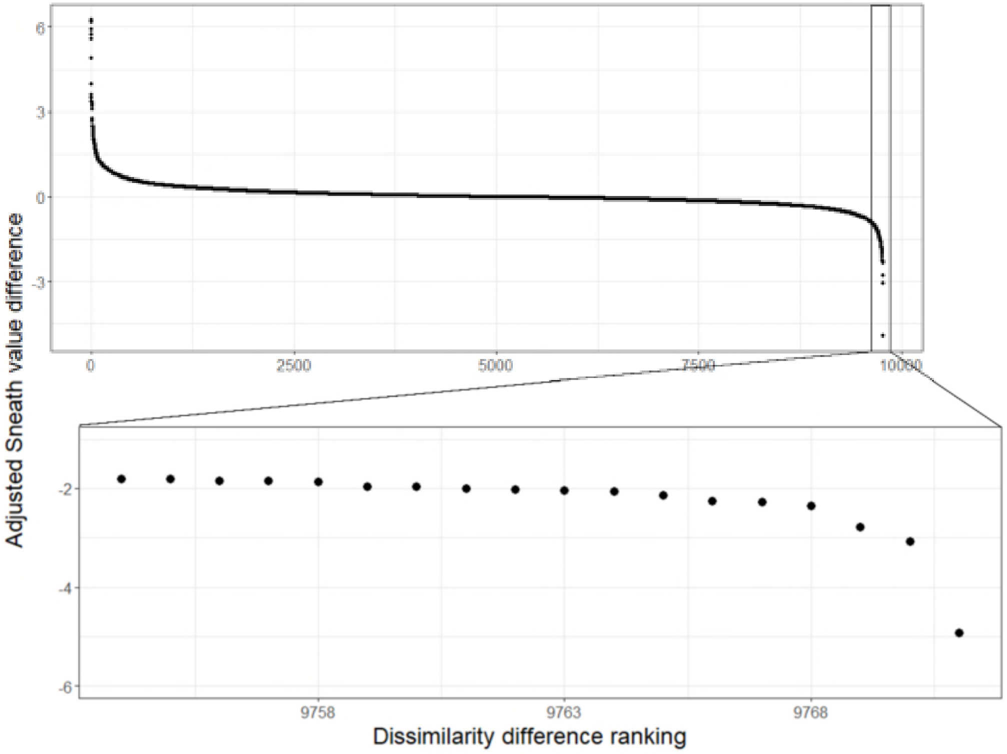

Fig. S2 Dissimilarity difference ranking for all 9,771 sand rat proteins against the difference in adjusted Sneath value compared to the murid homologue. The last 18 ranked proteins are enlarged in the bottom plot.

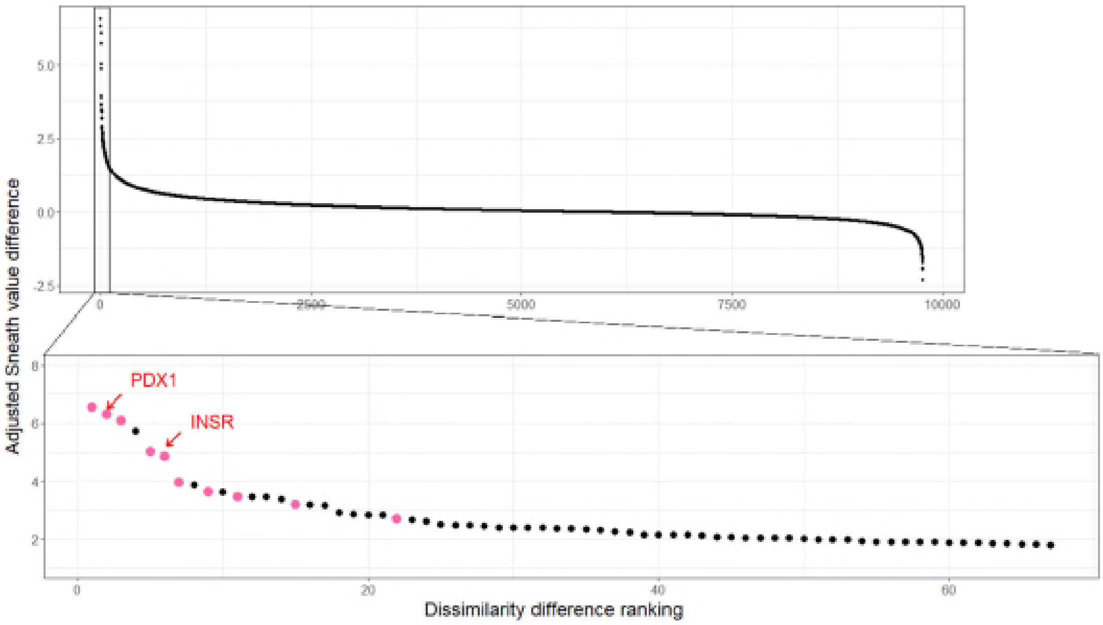

Fig. S3 Dissimilarity difference ranking for all 9771 sand rat proteins against the difference in adjusted Sneath value compared to the average of five species in the mouse-related clade (kangaroo rat (*Dipodomys ordii*), jerboa (*Jaculus jaculus*), blind mole rat (*Nannospalax galili*), mouse and rat). The top 67 ranked proteins are enlarged in the bottom plot with proteins PDX1 (rank 1) and INSR (rank 6) marked with an arrow. Amongst these proteins, those encoded by genes in the extreme GC-rich region are shown in pink.

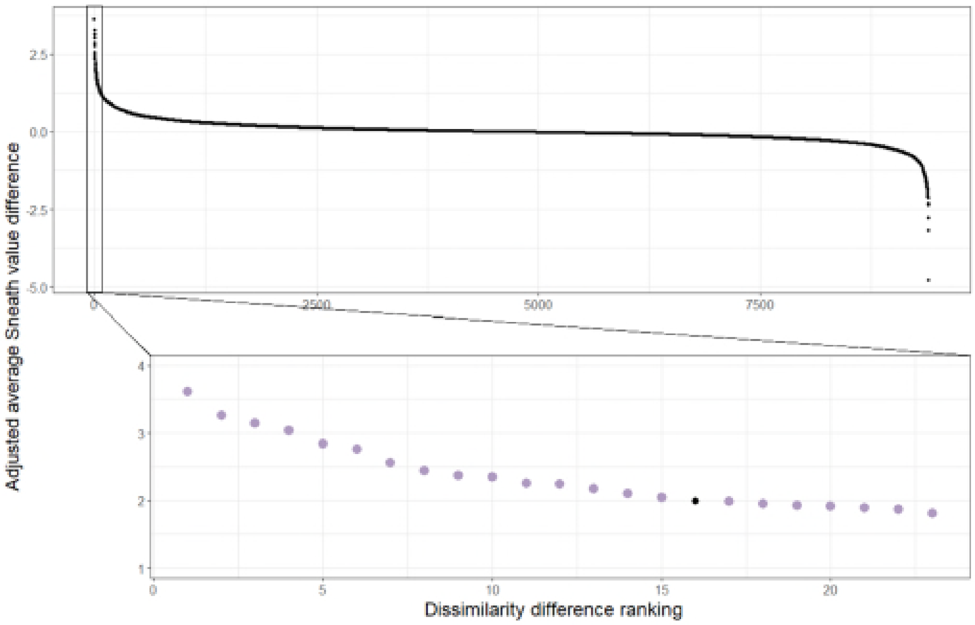

Fig. S4 Dissimilarity difference ranking for all 9403 gerbil proteins, comparing the average adjusted Sneath value of both gerbil species against the difference in average adjusted Sneath value of mouse and rat. The top 23 ranked proteins are enlarged in the bottom plot with proteins that are also aberrantly divergent in the sand rat shown in grey.

<u>Section 3: Association between aberrantly divergent proteins, high GC islands, and selection</u>

A. High GC regions in the Mongolian jird genome (1 Mbp window, 0.25 Mbp step)			
**Chromosome**	**Start**	**End**	**Length (bp)**
1	38,735,985	41,485,985	2,750,000
1	191,735,985	192,735,985	1,000,000
3	84,269,840	85,269,840	1,000,000
5	104,917,342	107,167,342	2,250,000
5	136,167,342	137,917,342	1,750,000
5	*145,128,769*	*149,762,599*	*4,500,000*
6	29,118,273	30,368,273	1,250,000
6	146,618,273	147,618,273	1,000,000
7	23,720,729	25,720,729	2,000,000
7	30,970,729	31,970,729	1,000,000
8	*3,150,922*	*3,681,255*	*500,000*
8	125,200,606	126,700,606	1,500,000
9	121,297,555	122,797,555	1,500,000
11	69,541,271	71,291,271	1,750,000
12	117,314,511	119,064,511	1,750,000
14	120,701,122	122,701,122	2,000,000
15	100,271,842	101,521,842	1,250,000
16	94,103,884	96,353,884	2,250,000
18	6,101,319	7,101,319	1,000,000

**B. High GC regions in the sand rat genome (1 Mbp window, 0.25 Mbp step)**			
**Chromosome**	**Start**	**End**	**Length (bp)**
1	38,735,985	39,735,985	1,000,000
1	39,985,985	41,485,985	1,500,000
1	191,735,985	192,735,985	1,000,000
3	84,269,840	85,269,840	1,000,000
5	104,917,342	107,417,342	2,500,000
5	136,167,342	137,917,342	1,750,000
5	*145,128,769*	*149,762,599*	*4,500,000*
6	29,368,273	30,618,273	1,250,000
6	146,618,273	147,618,273	1,000,000
7	23,720,729	26,220,729	2,500,000
8	*3,150,922*	*3,681,255*	*500,000*
8	125,200,606	126,700,606	1,500,000
9	121,297,555	123,047,555	1,750,000
11	69,541,271	71,291,271	1,750,000
12	117,314,511	119,064,511	1,750,000
14	120,951,122	122,701,122	1,750,000
15	99,771,842	101,521,842	1,750,000
15	101,771,842	102,771,842	1,000,000
16	94,103,884	96,353,884	2,250,000

Table S1 List of high GC regions used for analysis defined using sliding window analysis of average dS_{ws} and direct comparison of gene coding sequence (3,5). These locations are mapped using mouse chromosome coordinates. Most regions are obtained from dS_{ws} outlier analysis, with the exceptions being regions in italics, which are obtained from gene coding sequence comparison focusing on the ParaHox region and surrounding genes. (A) High GC regions in the Mongolian jird genome. (B) High GC regions in the sand rat genome.

Gene location<br><br>Protein type	High GC	Not high GC	Total
**Aberrantly divergent**	7	43	50
**Normal**	314	9705	10019
**Total**	321	9748	10069

Table S2 Summary of aberrantly divergent Mongolian jird proteins and whether their corresponding genes are in high GC peaks. Fisher's Exact Test reports p-value = 0.00096, estimated odds ratio = 5.03.

Gene type<br><br>Protein type	dS_{sw} outlier	Not dS_{sw} outlier	Total
**Aberrantly divergent**	19	31	50
**Normal**	45	9676	9721
**Total**	64	9707	9771

Table S3 Summary of aberrantly divergent sand rat proteins and whether their corresponding genes are dS strong to weak outliers. Fisher's Exact Test reports p-value = 4.4e-30, with an estimated odds ratio of 130.70.

Gene type<br><br>Protein type	dS_{ww} outlier	Not dS_{ww} outlier	Total
**Aberrantly divergent**	16	34	50
**Normal**	106	9615	9721
**Total**	122	9649	9771

Table S4 Summary of aberrantly divergent sand rat proteins and whether their corresponding genes are dS weak to weak outliers. Fisher's Exact Test reports p-value = 4.4e-19, with an estimated odds ratio of 42.58.

Gene type<br><br>Protein type	dS_{ss} outlier	Not dS_{ss} outlier	Total
**Aberrantly divergent**	15	35	50
**Normal**	29	9692	9721
**Total**	44	9727	9771

Table S5 Summary of aberrantly divergent sand rat proteins and whether their corresponding genes are dS strong to strong outliers. Fisher's Exact Test reports p-value = 8.8e-25, with an estimated odds ratio of 142.16.

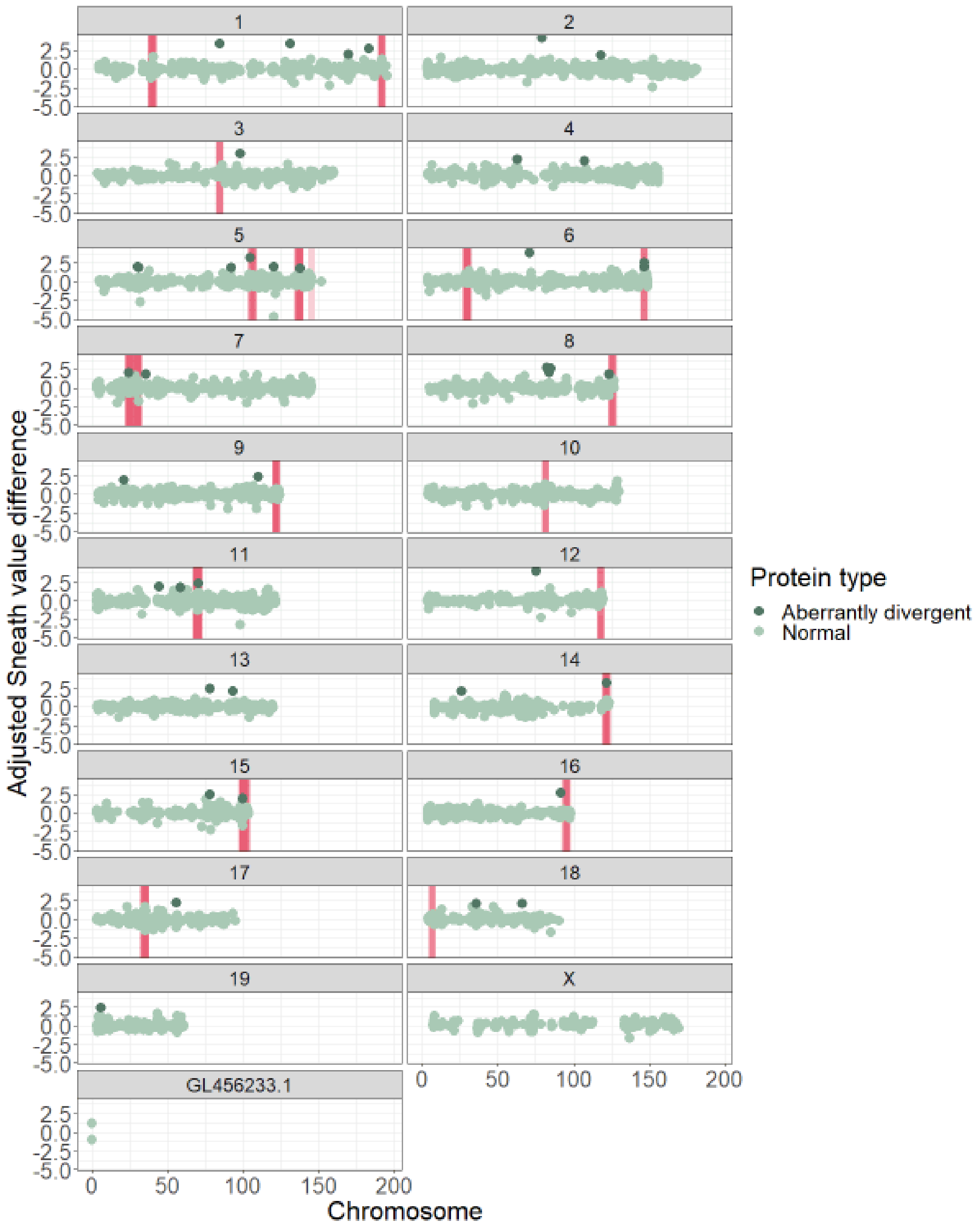

Fig. S5 Aberrantly divergent Mongolian jird proteins are frequently encoded by genes in GC-rich islands. Each panel shows one mouse chromosome, to which the locations of Mongolian jird orthologues are mapped. All analyzed Mongolian jird genes are displayed as dots plotted according to the midpoint position of their corresponding mouse orthologue. The position of each dot on the y-axis shows the difference in adjusted Sneath value between the Mongolian jird and mouse orthologues. Pink lines indicate locations of GC-rich regions identified previously (3,5).

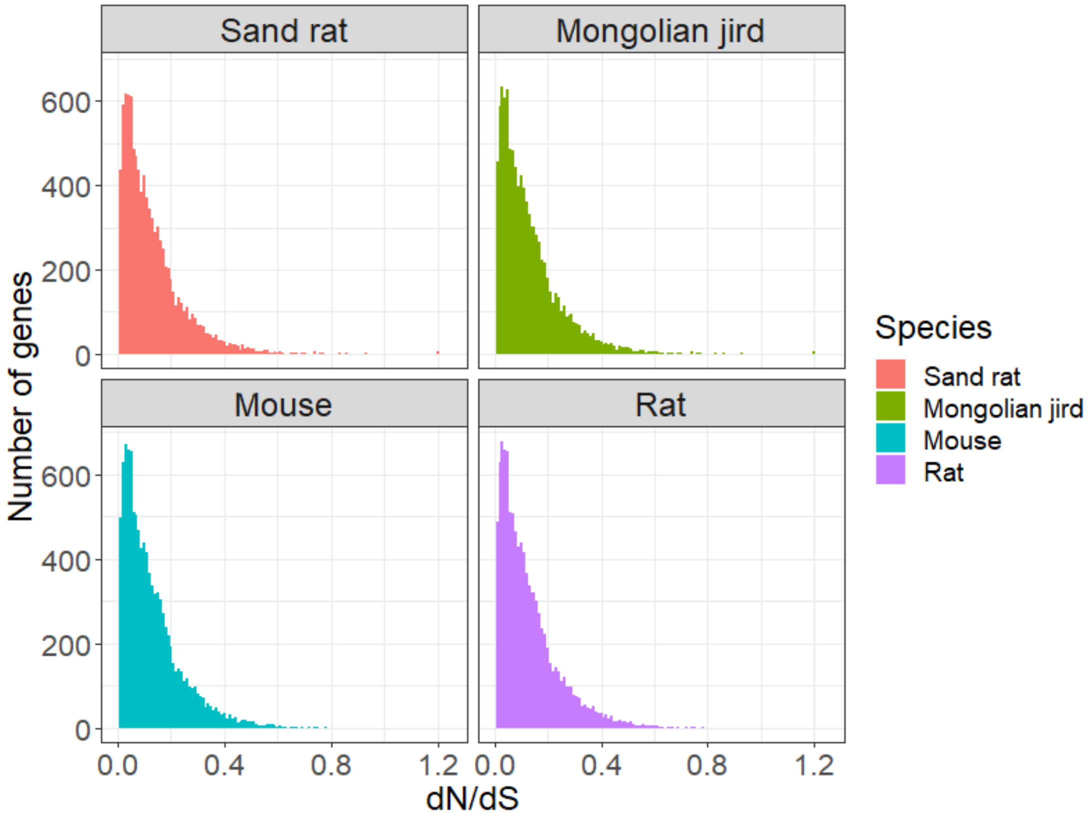

Fig. S6 Genome-wide distribution of dN/dS in gerbils and murids. Due to constraints in plot width, we have artificially converted the dN/dS values of five sand rat genes and five Mongolian jird genes with dN/dS > 1.2 to 1.2. The skewness of distribution was calculated for all four species, with sand rat skewness = 3.60, Mongolian jird skewness = 3.51, mouse skewness = 1.54, rat skewness = 1.52.

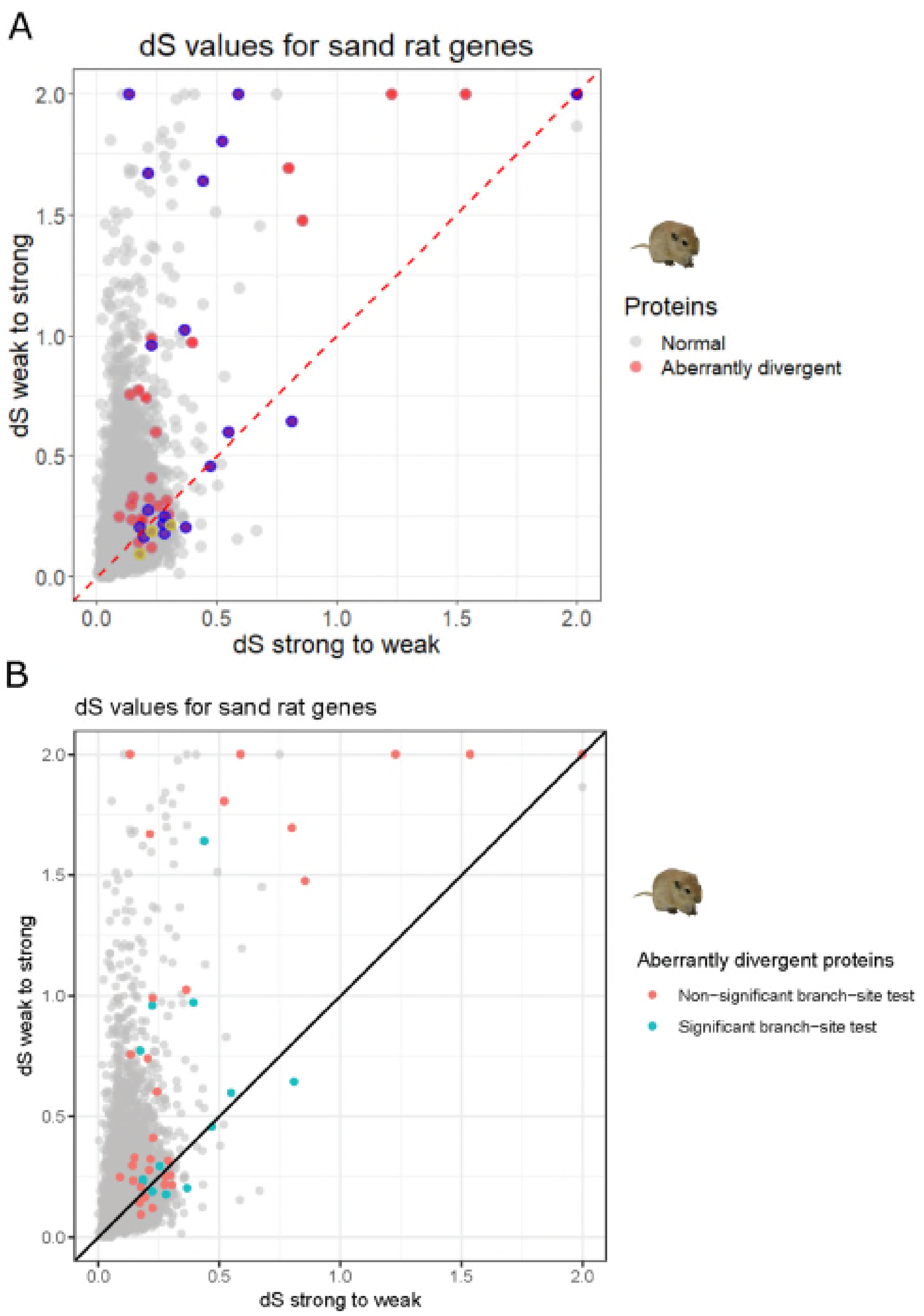

Fig. S7 Relation between aberrantly divergent sand rat proteins and evidence of positive selection. (A) Three genes encoding aberrantly divergent proteins under positive selection (dN/dS > 1) predicted by the branch test model in PAML are shown in yellow. Eighteen aberrantly divergent genes with dN/dS < 1 but with higher dN/dS in the gerbil lineage compared to the background rodent lineage are shown in purple. (B) Sand rat genes analyzed using the Godon program (36). Genes encoding aberrantly divergent proteins are shown in color, with genes that are unlikely to be under positive selection shown in red. The 13 aberrantly divergent genes with signs of positive selection at some sites are shown in green. These are *Cblc*, *Tex37*, *Mia3*, *Zfp3*, *Ppp1r3e*, *Ift46*, *Zfp105*, *Dmp1*, *Fbxo28*, *Nhlrc3*, *Kcp*, *Phyh*, and *Ifngr2*.

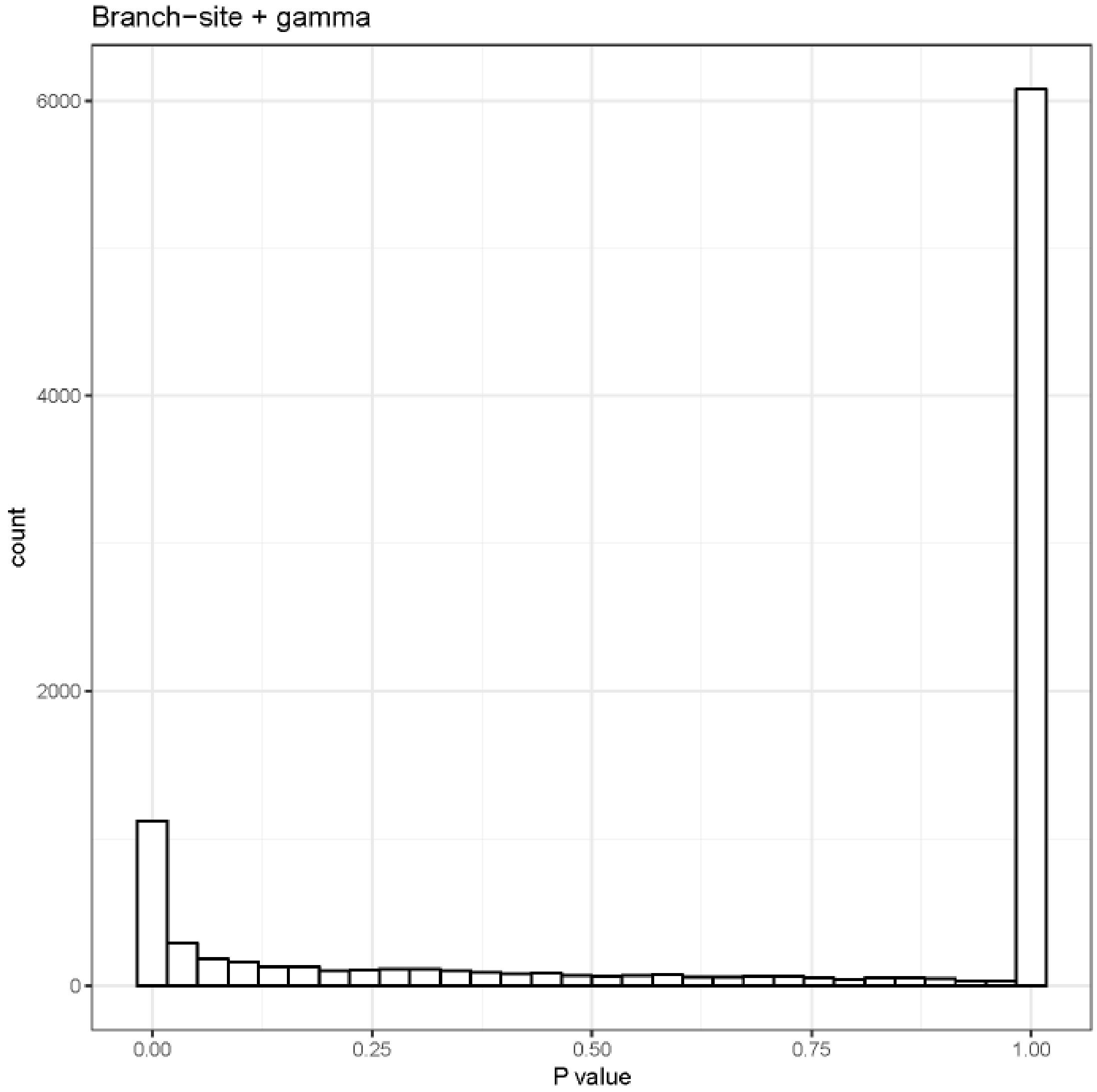

Fig. S8 Distribution of p-values generated by the Godon program (36). All sand rat genes were analyzed using the branch-site model with codon gamma rate variation and p-values were generated with likelihood ratio tests (LRT) between the null and alternative models.

Section 4: Curation and alignment of 1-to-1 orthologues

Species name	Common name	genome-build	genome-date	genome-build-accession	genebuild-last-updated
Cavia porcellus	Domestic guinea pig	Cavpor3.0	2008-03	NCBI:GCA_000151735.1	2017-07
Chinchilla lanigera	Long-tailed chinchilla	ChiLan1.0	2012-05	NCBI:GCA_000276665.1	2017-07
Dipodomys ordii	Ord's kangaroo rat	Dord_2.0	2014-12	NCBI:GCA_000151885.2	2017-07
Fukomys damarensis	Damara mole rat	DMR_v1.0	2014-09	NCBI:GCA_000743615.1	2017-02
Homo sapiens	Human	GRCh38.p12	2013-12	NCBI:GCA_000001405.27	2018-07
Ictidomys tridecemlineatus	Thirteen-lined ground squirrel	SpeTri2.0	2011-11	NCBI:GCA_000236235.1	2017-07
Jaculus jaculus	Lesser Egyptian jerboa	JacJac1.0	2012-07	NCBI:GCA_000280705.1	2017-02
Meriones unguiculatus	Mongolian gerbil	MunDraft-v1.0	2017-06	NCBI:GCA_002204375.1	2018-12
Mus musculus	Mouse	GRCm38.p6	2012-01	NCBI:GCA_000001635.8	2018-09
Nannospalax galili	Upper Galilee mountains blind mole rat	S.galili_v1.0	2014-05	NCBI:GCA_000622305.1	2017-02
Octodon degus	Degu	OctDeg1.0	2012-05	NCBI:GCA_000260255.1	2017-07
Rattus norvegicus	Rat	Rnor_6.0	2014-07	NCBI:GCA_000001895.4	2017-01

Table S6 Accession IDs of predicted genome annotations used in analysis. These genome annotations were previously used to identify GC-rich regions in the gerbil genome (5).

References

Citations refer to references in main manuscript.
